# The temporal cost of deploying attention limits accurate target identification in rapid serial visual presentation

**DOI:** 10.1038/s41598-023-30748-z

**Published:** 2023-03-03

**Authors:** Anna R. Kimata, Bryan Zheng, Takeo Watanabe, Wael F. Asaad

**Affiliations:** 1 Department of Neuroscience, The Carney Institute, Brown University, Providence, RI USA; 2 The Warren Alpert Medical School of Brown University, Providence, RI USA; 3 Department of Neurosurgery, Brown University Alpert Medical School and Rhode Island Hospital, 593 Eddy Street, Providence, RI 02903 USA; 4 Norman Prince Neurosciences Institute, Rhode Island Hospital, Providence, RI USA; 5 Department of Cognitive, Linguistic, and Psychological Sciences, Brown University, Providence, RI USA

**Keywords:** Attention, Sensory processing

## Abstract

Lag-1 sparing is a common exception to the attentional blink, where a target presented directly after T1 can be identified and reported accurately. Prior work has proposed potential mechanisms for lag 1 sparing, including the boost and bounce model and the attentional gating model. Here, we apply a rapid serial visual presentation task to investigate the temporal limitations of lag 1 sparing by testing three distinct hypotheses. We found that endogenous engagement of attention to T2 requires between 50 and 100 ms. Critically, faster presentation rates yielded lower T2 performance, whereas decreased image duration did not impair T2 detection and report. These observations were reinforced by subsequent experiments controlling for short-term learning and capacity-dependent visual processing effects. Thus, lag-1 sparing was limited by the intrinsic dynamics of attentional boost engagement rather than by earlier perceptual bottlenecks such as insufficient exposure to images in the stimulus stream or visual processing capacity limitations. Taken together, these findings support the boost and bounce theory over earlier models that focus only on attentional gating or visual short-term memory storage, informing our understanding of how the human visual system deploys attention under challenging temporal constraints.

## Introduction

Looking into the woods you notice a shifting of branches or rustling of leaves. Attending to that location more intently now allows identification of the first target and the quick recognition of the creature causing those movements (second target). This has potential survival value, whether predator or prey. Thus, the rapid identification of novel visual features within the environment is highly adaptive. Subjectively, directing attention towards and subsequently identifying specific stimuli is instantaneous. However, this experience is limited by underlying perceptual processes that control how visual information is sampled and analyzed by the brain. To study the mechanisms governing the deployment of attention, prior work has used rapid serial visual presentation (RSVP) paradigms^1^. These tasks entail presenting a rapid stream of stimuli (e.g., alphanumeric characters, symbols, etc.) that contain one or more cued targets that subjects must identify and report. Findings from such studies have demonstrated that top-down selective attention is critical for rapid deployment of attention towards features that match a subject’s template for target stimuli^2–4^.

A well-studied phenomenon in RSVP paradigms is the attentional blink (AB)^5–10^. First reported by Broadbent and Broadbent^5^, the AB describes the inability to accurately identify a second target (T2) in a stream of stimuli if T2 is presented within 500 ms of target 1 (T1). Lag 1 sparing*,* which occurs when T2 is presented immediately after T1, is a known exception to the AB that allows for accurate report of both targets^11^, although the two targets are often perceived in the incorrect order (order reversal)^12^.

Prior work probing the mechanisms that underlie lag 1 sparing have yielded conflicting theories to explain this phenomenon. Expanding on limit-capacity AB models^6^, several groups have suggested that lag 1 sparing occurs because T1 triggers the opening of an attentional gate. The closing of this gate is delayed such that a T2 presented sufficiently close to T1 allows both targets to be processed together in the same attentional episode^13–16^. More recently, the demonstration of extended sparing (accurate identification of stimuli beyond T2) has led to the “boost and bounce theory,” which posits that T1 generates an attentional boost that assists the processing of T2 and other temporally proximal stimuli^17^.

Despite this prior work, it remains unclear how the temporal dynamics of stimulus presentation impact endogenous control of attention in lag 1 sparing. Other groups have shown that stimulus type, task goal, and expected probability of target appearance impact target accuracy in lag 1 sparing^18–22^. Several of these studies have also supported the idea that endogenous control of the attentional window may directly modulate the ability to accurately identify successive targets^20,21^. Additionally, one prior study suggested that attentional facilitation from the identification of target 1 has a temporal delay^23^; however, these temporal dynamics of the lag 1 phenomenon have not been sufficiently explored. Therefore, using a modified, single-stream version of Potter and Levy’s RSVP paradigm, we attempt to dissect the potential critical features of this task to better understand lag 1 sparing, focusing on the fundamental temporal dynamics of attention in this context, and further assessing the potential contributions of rapid learning and visual processing capacity.

Prior studies have investigated temporal dynamics of attention in RSVP. Several groups have looked at the time course of attentional capture using a cue-target format. These studies have typically used “pop-out” cue stimuli, thus probing bottom-up, rather than endogenous, top-down activation of attention^6,13,24,25^. Furthermore, many prior RSVP and lag-1 sparing studies presented alphanumeric characters, words, or shapes in their RSVP streams^4,5,9,10,20,21,26,27^. This use of lexical or simple geometric stimuli yields information on how familiar or perhaps overlearned symbols are visually processed and identified, and so may not be fully representative of attentional dynamics in other circumstances^28^. Specifically, the extent to which endogenous processing dynamics are similar between lexical, iconic, and naturalistic stimuli is unclear. Our present study addresses these gaps by implementing a single stream RSVP design where all stimuli (including the cue or “T1” and target, “T2”) are novel images within the RSVP sequence, and so may not have a firmly pre-established neural representation to guide detection and identification. This modified task allows us to study the temporal dynamics of the lag-1 window of attention. Using this paradigm, we show that attentional engagement in lag 1 sparing requires approximately 50–100 ms, supporting the boost and bounce theory of lag-1 sparing. Furthermore, we observe and quantify a longer latency for the engagement of top-down versus bottom-up attention.

## Results

**Figure 1:**
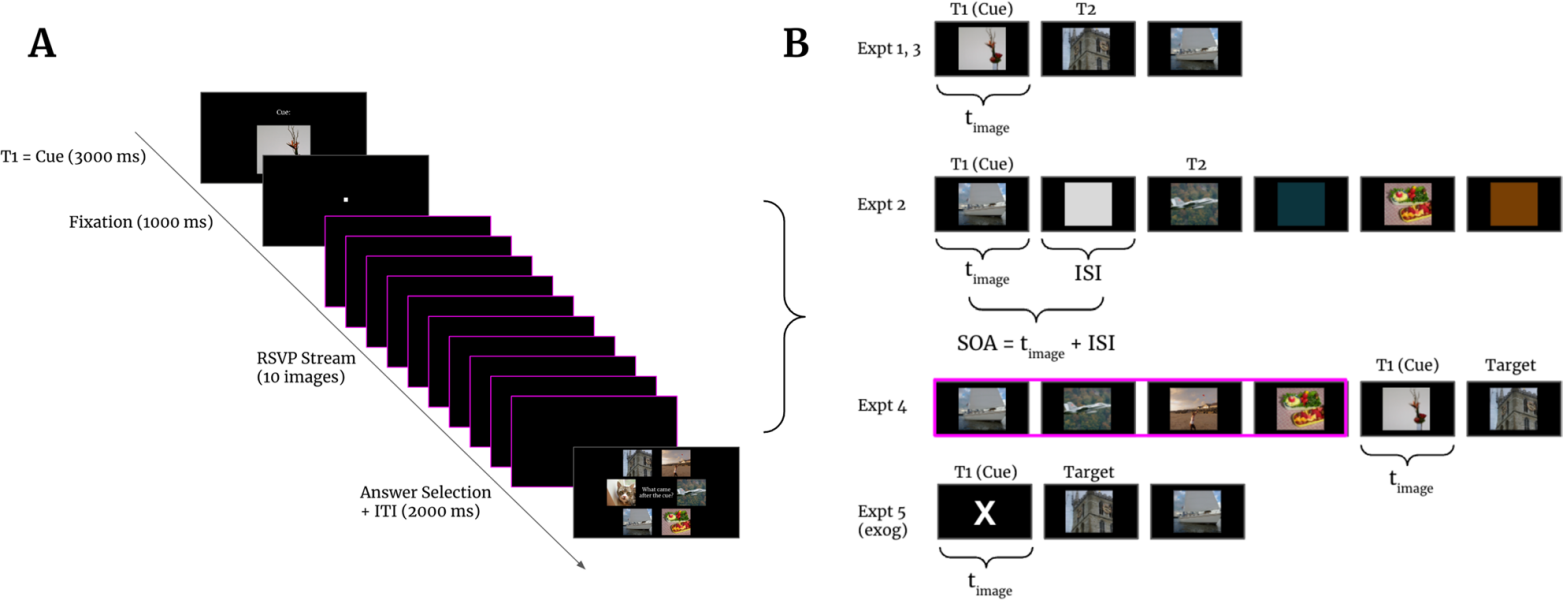
Illustrates stimulus and trial structure for experiments 1–5. (**A**) Initial T1 cue (6.54°, 300 × 300 pixels) presentation (3000 ms) was followed by a 1000 ms fixation, 10 images in a RSVP stream, an answer selection screen, and a 2000 ms ITI. (**B**) Sample of 3 images from the 10-image RSVP stream to illustrate stimulus structure for each experiment. All trials for all experiments always had 10 images. T2 image directly followed the T1 cue image, and T1 and T2 stimuli were always presented within the 10-image RSVP sequence. Experiment 1, Experiment 3, and Experiment 5, SOA = t_image_ and no fillers were present_._ Experiments 2A-C used fillers and varied SOA and t_image_ according to the specified conditions (see Table 1; SOA = t_image_ + ISI). In Experiment 4, the target was always preceded by the same four images (SOA = t_image_). The exogenous T1 cue in experiment 5 was a large white “X”, and the endogenous (top-down) T1 cue was identical to the Experiment 1 and 3 conditions.

Through a series of 5 experiments, we used a modified version of the RSVP paradigm to probe the dynamics of attentional facilitation in lag-1 sparing. Our task required subjects to identify, within a single 10-image RSVP stream, a trial-unique cue image (T1), and then encode the image (T2) immediately following that cue for later selection at the end of each trial (see Task in “Methods” section, Fig. 1). The T1 image was presented separately, prior to the RSVP stream, to inform subjects of its identity, and then again within the stream immediately prior to T2. Because T2 was always presented immediately after T1, lag-1 sparing allowed, under certain conditions, subjects to detect and encode T2 for later report. By systematically modifying those conditions, we sought to test three hypotheses concerning the underlying mechanisms of lag-1 sparing: (1) Presentation rate may limit lag-1 sparing because sufficient time is needed between T1 and T2 for attentional facilitation to engage in order to achieve accurate T2 identification; (2) Accurate identification of T2 is limited by the duration of image presentation such that insufficient experience with the T1 or T2 images in the RSVP stream prevents the formation of suitable representations for working memory (note that working memory is needed for both T1 and T2: T1 must be compared against the contents of working memory to initiate attentional processes to capture T2, which itself must be converted to a working memory representation for 6-alternative forced choice at the end of the trial). (3) Alternatively, presentation rate may limit lag-1 sparing due to visual capacity limitations such that visual processing could be saturated in such a manner as to interfere with attentional deployment. We found that out of these three competing hypotheses, the data supported the hypothesis that endogenous attentional facilitation in lag-1 sparing is limited by presentation rate, such that a sufficient temporal delay to “boost” processing of the T2 image is necessary for high performance.

### Experiment 1: a temporal delay is needed for T2 attentional capture in lag 1 sparing

**Figure 2:**
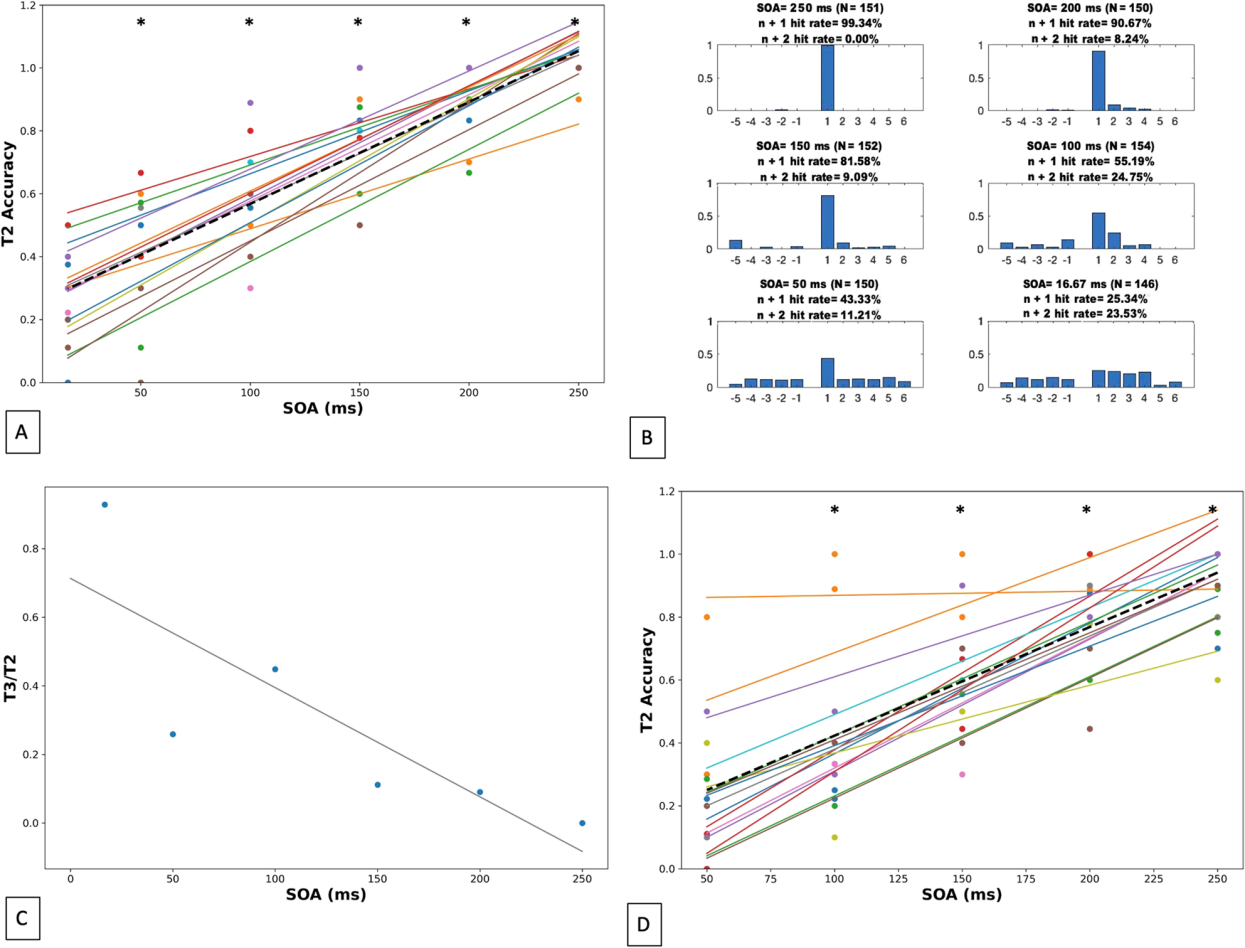
Temporal threshold for lag 1 sparing lies between 50 and 100 ms. Asterisks (*) indicate T2 accuracy was significantly greater than chance (0.1667). (**A**) Variation of presentation rate and image duration (uncontrolled condition). Average T2 (lag 1) accuracy for each SOA [16.67–250 ms] across 16 subjects shows increasing accuracy as the SOA increases. Linear fit for each individual subject is shown by the solid-colored lines (range of slops: [0.0021, 0.0044]). Group effect shown by the dashed black line (slope = 0.0032). Only T2 accuracy at SOA = 16.67 ms were not significantly different than chance (*p* = 0.052). (**B**) Average T2 accuracy (y-axis) by distance from the target in the RSVP stream (x-axis) for each SOA. Number of trials represented by the data in each subplot is shown in parenthesis. (**C**) Ratio of n + 2/n + 1 hit rates across SOAs demonstrates an increase in the proportion of T3 errors relative to T2 as presentation rates increase. Grey line represents linear regression fit to plotted data points. (**D**) Variation of presentation rate while controlling for image duration. Average T2 (lag 1) accuracy for each SOA [50-250 ms] across 16 subjects on trials shows increasing accuracy as the SOA increases. Linear fit for each individual subject is shown by the solid-colored lines (range of slopes: [0.0026, 0.0052]). Group effect shown by the dashed black line (slope = 0.0035). T2 accuracy at 50 ms SOA was not significantly different than chance (*p* = 0.187).

To investigate how quickly top-down attention can be engaged to reliably process the T2 stimulus, we systematically varied the speed of RSVP image presentation (SOA = [16.67, 50, 100, 150, 200, 250 ms]; ISI = 0 ms; Table 1). In experiment 1, subjects performed 10 trials at each SOA, for a total of 60 trials. Analysis of accuracy, defined as the percentage of trials where participants reported T2 correctly, demonstrated that T2 accuracy decreased linearly as SOA shortened from 250 to 16.67 ms in both individual and group analyses (Individual slope range: [0.0021, 0.0044]). Group effect: T2 accuracy = 0.0032 * SOA + 0.24; Fig. 2A,B). Performance on trials with 16.67 ms SOA was not significantly different than chance (*p* = 0.052), while performance on 50 ms SOA trials was significantly greater than chance (*p* < 0.001; Fig. 2A). The ratio of n + 2/n + 1 hit rates also increased for faster presentation rates (Fig. 2C). Pre-pattern intrusion errors were not seen for the 150, 200, or 250 ms conditions, indicating that these SOAs may provide sufficient time to deploy an attentional boost while also maintaining correct order perception (Fig. 2B). An identical experimental structure using alphanumeric characters produced a similar trend (T2 accuracy = 0.0031 * SOA + 0.26; Supplementary Figs. 3–4), suggesting that the temporal dynamics of lag 1 sparing with naturalistic scenes are not appreciably different than with lexical or iconic stimuli.

**Table 1:** SOA, filler, and presentation feature details for main and supplemental blocks.

Experiment	t_image_ (ms)	ISI (ms)	SOA (ms) = t_image_ + ISI	Trials per block	Exogenous versus endogenous cue	Stimulus type	Figures
1	16.67, 50, 100, 150, 200, 250	0	16.67, 50, 100, 150, 200, 250	60	Endogenous	Images	2
2A	50	0, 50, 100, 150, 200	50, 100, 150, 200, 250	50	Endogenous	Images	2D, 3, S1A
2B	50, 100, 150, 200, 250	200, 150, 100, 50, 0	250	50	Endogenous	Images	3, S1B
2C	16.67, 33.34, 50	33.34, 16.67, 0	50	30	Endogenous	Images	S2
3A-B, 4	50	0	50	30	Endogenous	Images	4
5	16.67, 50, 66.67, 83.34, 100, 116.67, 134.34, 150, 166.67, 200	0	16.67, 50, 66.67, 83.34, 100, 116.67, 134.34, 150, 166.67, 200	90	Exogenous and endogenous	Images	5
Supp 1	16.67, 50, 100, 150, 200, 250	0	16.67, 50, 100, 150, 200, 250	60	Endogenous	Alphanumeric	S3A
Supp 2A	50	0, 50, 100, 150, 200	50, 100, 150, 200, 250	50	Endogenous	Alphanumeric	S3B
Supp 2B	50, 100, 150, 200, 250	200, 150, 100, 50, 0	250	50	Endogenous	Alphanumeric	S3C
Acclimation	250	0	250	10	Endogenous	Images	1

### Experiment 2: the temporal dynamics of lag-1 sparing are limited by rate of stimulus presentation, not stimulus duration

**Figure 3:**
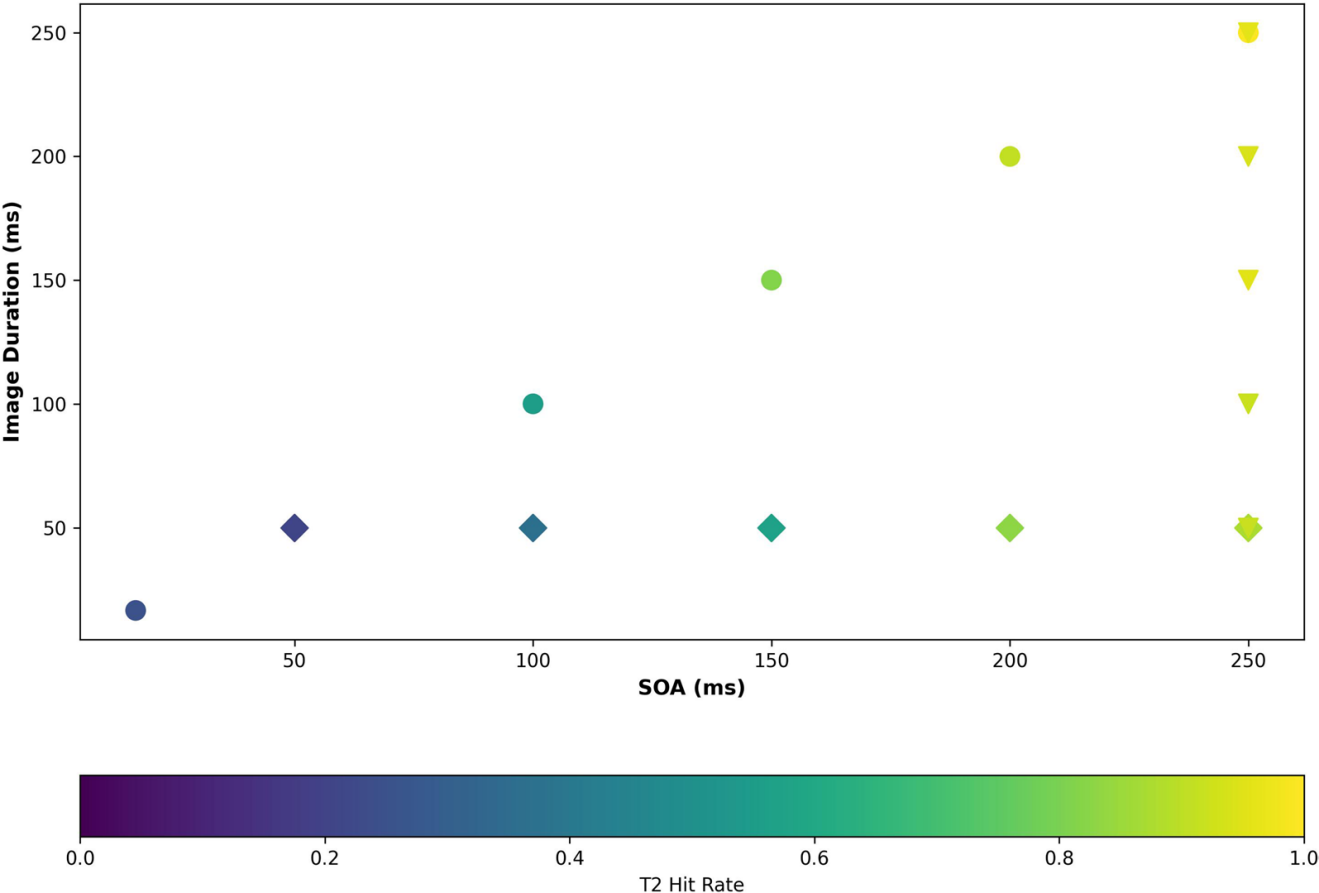
Relationship between presentation rate (SOA), Image Duration (t_image_), and T2 (lag 1) Accuracy. Circles represent data from Experiment 1. Diamonds represent data from Experiment 2A. Inverted triangles represent data from Experiment 2B. Presentation rate (SOA), not accumulation of visual information (image duration) defines the lower limit of endogenous boost in attention to lag-1 targets.

In experiment 2, we parametrically tested the factors that may contribute to the temporal delay in lag-1 attentional facilitation in two separate blocks (2A and 2B; see Table 1 for trial details). In experiment 2A, image duration was fixed at 50 ms (t_image_ = 50 ms), and the SOA varied from 50 to 250 ms. The interstimulus intervals (ISIs) therefore ranged from 0 to 200 ms (ISI = 0–200 ms; Fig. 1). Thus, this experiment tested hypothesis 1 by varying presentation rate while controlling for stimulus duration. To control for low-level visual stimulation, filler squares of equal size as the images were displayed between stimuli during the ISI (Fig. 1; Methods-“Experimental setup” section; note these “fillers” were not intended to function as masks). The results of Experiment 2A demonstrated that, even with a 50 ms image duration, performance could nonetheless be quite high with longer SOAs (over 80% accuracy selecting T2 correctly; Fig. 3, Supplementary Fig. 1A). Performance decreased as SOAs decreased, demonstrating presentation rate, rather than image duration, was the limiting factor in the attentional performance observed in Experiment 1 (Individual slope range: [0.0026, 0.0052]; Group effect: T2 accuracy = 0.0035 * SOA + 0.08; Fig. 2D). The isolated impact of presentation rate in Experiment 2A extends the results of Experiment 1: performance accuracy declined with decreasing SOA. Further, we noted that at presentation rates of 50 ms, T2 accuracy was not significantly different than chance (*p* = 0.187; Fig. 2D, Supplementary Fig. 1A), while at presentation rates of 100 ms T2 accuracy was significantly greater than chance (*p* < 0.001). This indicates that the lower temporal limit for activation (“boost”) of attention in lag 1 sparing lies between 50 and 100 ms.

Next, in Experiment 2B, hypothesis 2 was tested: We used a fixed presentation rate (SOA = 250 ms) with a varying image duration (t_image_ = 50–250 ms, ISI = 250–t_image_, SOA = 250 ms) to assess the impact of image duration apart from presentation rate. Here, high T2 accuracy was observed in all conditions (mean = 93.88%, range = 91.50–96.08%; Fig. 3, Supplementary Fig. 1B). These data are consistent with the notion that, given a sufficiently slow presentation rate (SOA—250 ms) the ability to capture T2 was not significantly impacted by the duration of visual experience with each image. This observation held when alphanumeric characters were used instead of image stimuli (Supplemental Experiment 2A,B, Supplemental Fig. 3B,C).

We observed a similar pattern of results as Experiment 2A in Experiment 2C, where the SOA was held at 50 ms (rather than 250 ms in Experiment 2A) while image duration varied at lower values (t_image_ = 16.67 ms, 33.34 ms, or 50 ms; ISI = 50 − t_image_; conditions equally weighted). Here, T2 accuracy was not significantly different across these three conditions (one-way ANOVA: *p* = 0.7119; Supplementary Fig. 2). Overall accuracy was, expectedly, fairly low (t_image_ [50 ms] = 33.33%, t_image_ [33.34 ms] = 30.88%, t_image_ [16.67 ms] = 25.00%), attributable to the short SOA. These results further support hypothesis 1 by demonstrating that the rate of presentation (SOA) had the greatest impact on top-down attention engagement in the lag-1 sparing context.

### Experiment 3: learning does not improve T2 accuracy for higher presentation rates

**Figure 4:**
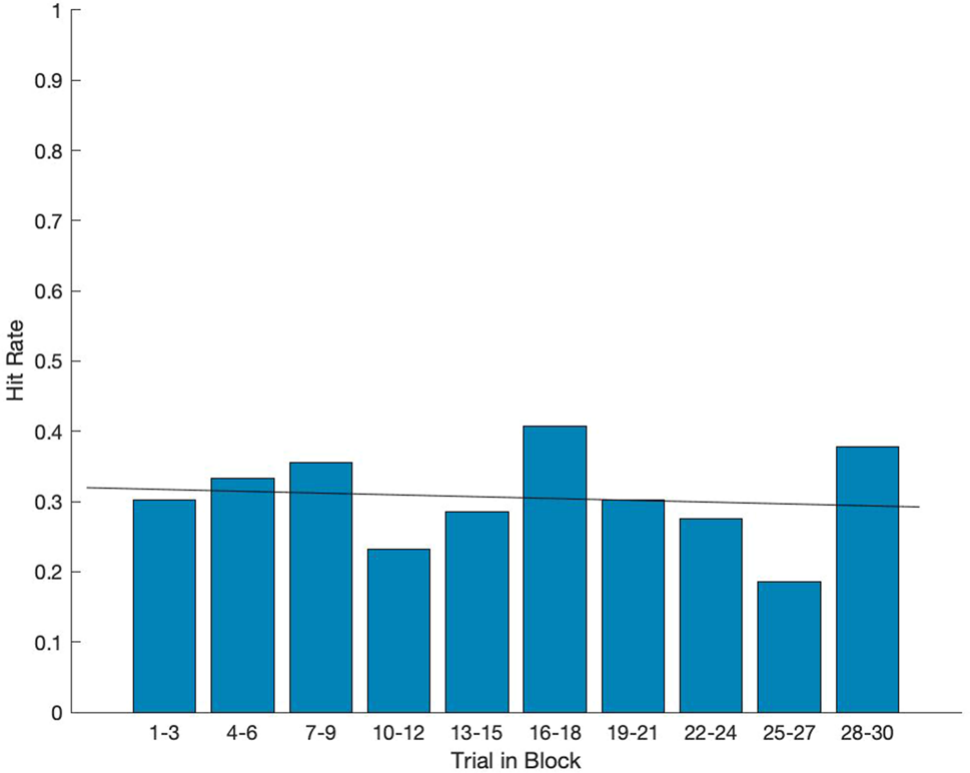
No learning observed over 30 repeated trials of 50 ms SOA. Experiment 3 average T2 accuracy (y-axis) across a bin of 3 consecutive trials (x-axis). A total of 10 bins are shown, representing results from all trials (1–30) of experiment 3 (N = 18 subjects). Best fit line (black) from least squares regression of target hit rates over the 30-trial block. Experiment 3 trials were identical to Experiment 1 in structure (SOA = t_image_, t_fill_ = 0 ms). At a below threshold range SOA, subjects are not able to perform with high accuracy, likely due to insufficient time to engage lag-1 facilitation.

Can subjects adapt attentional dynamics to improve T2 capture in the more challenging conditions? Experiment 3 consisted of 30 trials where the RSVP rate of presentation and image duration were both held constant at 50 ms (SOA = t_image_ = 50 ms). Analysis of average T2 accuracy over time demonstrated that performance did not improve across trials (Fig. 4). This suggests that at short SOAs, the ability to accurately identify the T2 stimulus was not subject to rapid learning or adaptation effects, though the potential benefit of more extensive experience (e.g., repeated sessions across days) was not tested.

### Experiment 4: the degradation of performance at faster presentation rates is not due to capacity limitations of visual processing

**Figure 5:**
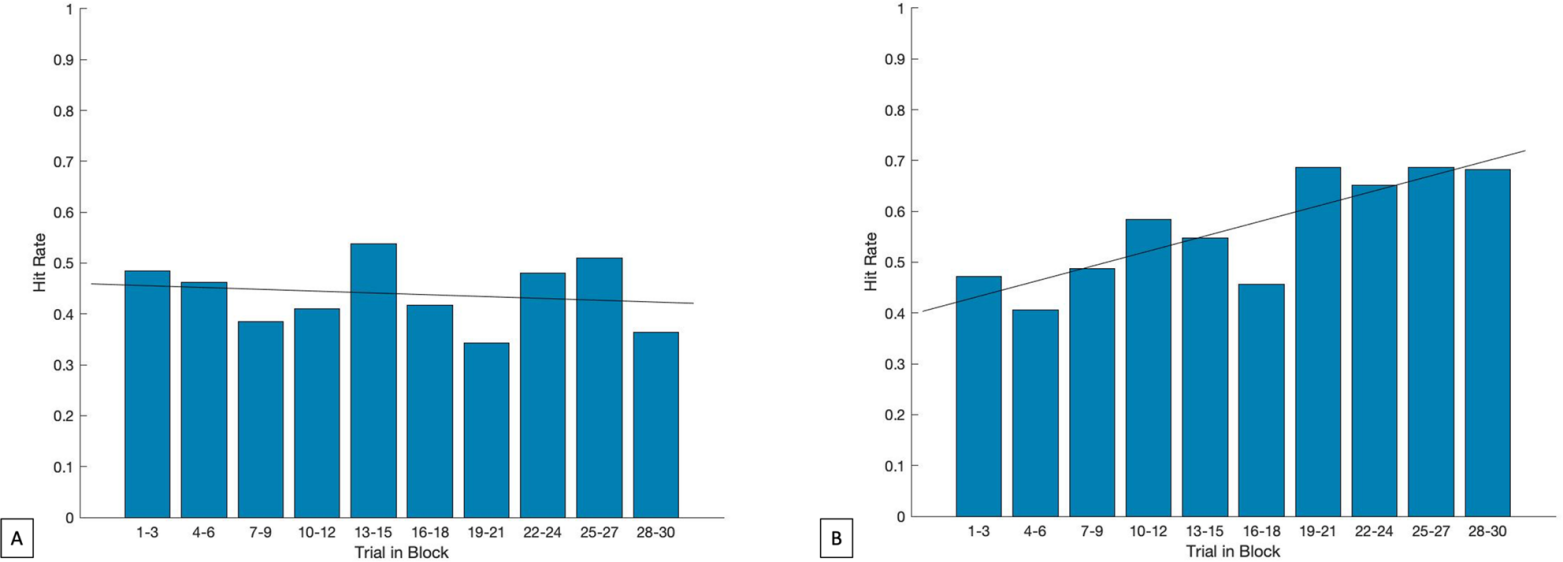
Lag-1 performance is not limited by capacity-dependent visual processing, but rather sufficient time to engage the T2 attentional boost. Average T2 hit rate (y-axis) across a bin of 3 consecutive trials (x-axis). A total of 10 bins are shown, representing results from all trials (1–30). Best fit line (black) from least squares regression of target hit rates over the 30-trial block. (**A**) Experiment 4 (unaware, N = 14 subjects), participants did not demonstrate learning when unaware of the sequence preceding the target. (**B**) Experiment 4 (aware, N = 13), participants demonstrated significant learning compared to the 50 ms SOA accuracy from Experiment 1 (*p* < 0.0001).

High presentation rates may limit T2 performance due to some aspect of the rapidly changing visual input rather than due to the constrained dynamics of attention itself (hypothesis 3). For example, in a capacity-limited processing model, the rapid appearance of new visual stimuli might saturate visual processing mechanisms and disrupt attentional engagement. In other words, the rapid influx of new visual stimuli, at a low level, may interfere with higher-order cognitive processes, unrelated to any intrinsic dynamics of those higher order functions. We explored this possible alternative explanation in Experiment 4. Here, we used a rapid presentation rate (t_image_ = SOA = 50 ms) but manipulated the image stream such that the same four images always preceded the T1. This design introduced a 250 ms delay between the onset of the first image in the sequence and the presentation of the T2 image but preserved the overall RSVP presentation rate (one image per 50 ms). If high performance is not impacted by capacity-limited processing, we expected that the “early warning” enabled by this fixed sequence would significantly improve T2 accuracy by providing 250 ms for attention facilitation mechanisms to engage. Conversely, if the rapidly changing visual inputs were a critical limiting factor, this manipulation should fail to improve performance. Results from experiment 4 demonstrated that, when participants were informed of the presence of a fixed sequence (that nonetheless varied in exact position within the RSVP stream: the initial stimulus in the sequence could occur in positions 2, 3, or 4), T2 accuracy improved from 25.00 to 71.43% (Fig. 5B)—a significantly higher maximum hit rate than observed for the relevant 50 ms SOA condition in Experiment 1 (*p* < 0.0001; Figs. 2 and 5B). Importantly, because the T2 could now occur in only a smaller subset of positions (positions 7, 8 or 9 in the visual stream), we confirmed that subjects were indeed using sequence-derived information rather than simply learning to focus attention on a smaller set of possible, later-appearing targets by running the same experiment but without informing them of the presence of a fixed-sequence predicting T2. Here, we found that when subjects were not informed of the sequence, target accuracy was not significantly different from the original 50 ms condition in Experiment 1 (*p* = 0.9717; Figs. 2 and 5A). These results support the notion that, at rapid presentation rates, accurate processing and identification of a lag-1 visual target is limited by the intrinsic dynamics of attentional facilitation rather than by early visual capacity limitations caused by the rapid appearance of successive stimuli. This finding also supports the concept of extended sparing^29^.

These data also served to rule out an alternative interpretation for experiment 2. Because manipulating Experiment 2B image durations (SOA = 250 ms, t_image_ = [50–250 ms]) involved trials where pictures in the RSVP stream were succeeded by ISI “fillers” (and thus no image presentation), it was possible that the higher performance observed in Experiment 2B may have been attributed simply to viewing fewer stimuli per 250 ms interval. In Experiment 4, because the cue was preceded by a constant sequence of four images, participants had 250 ms from the onset of the sequence to the target to engage attentional mechanisms but this interval was nonetheless filled with potentially interfering visual inputs. Thus, high T2 accuracy here (Fig. 5B) supports the idea that successful deployment of attentional engagement in Experiment 2B was the result of slow presentation rate and not confounded by a relative paucity of low-level visual stimulation.

### Experiment 5: boosts of top-down versus bottom-up attention in lag-1 sparing engage similar mechanisms but follow a different time course

**Figure 6:**
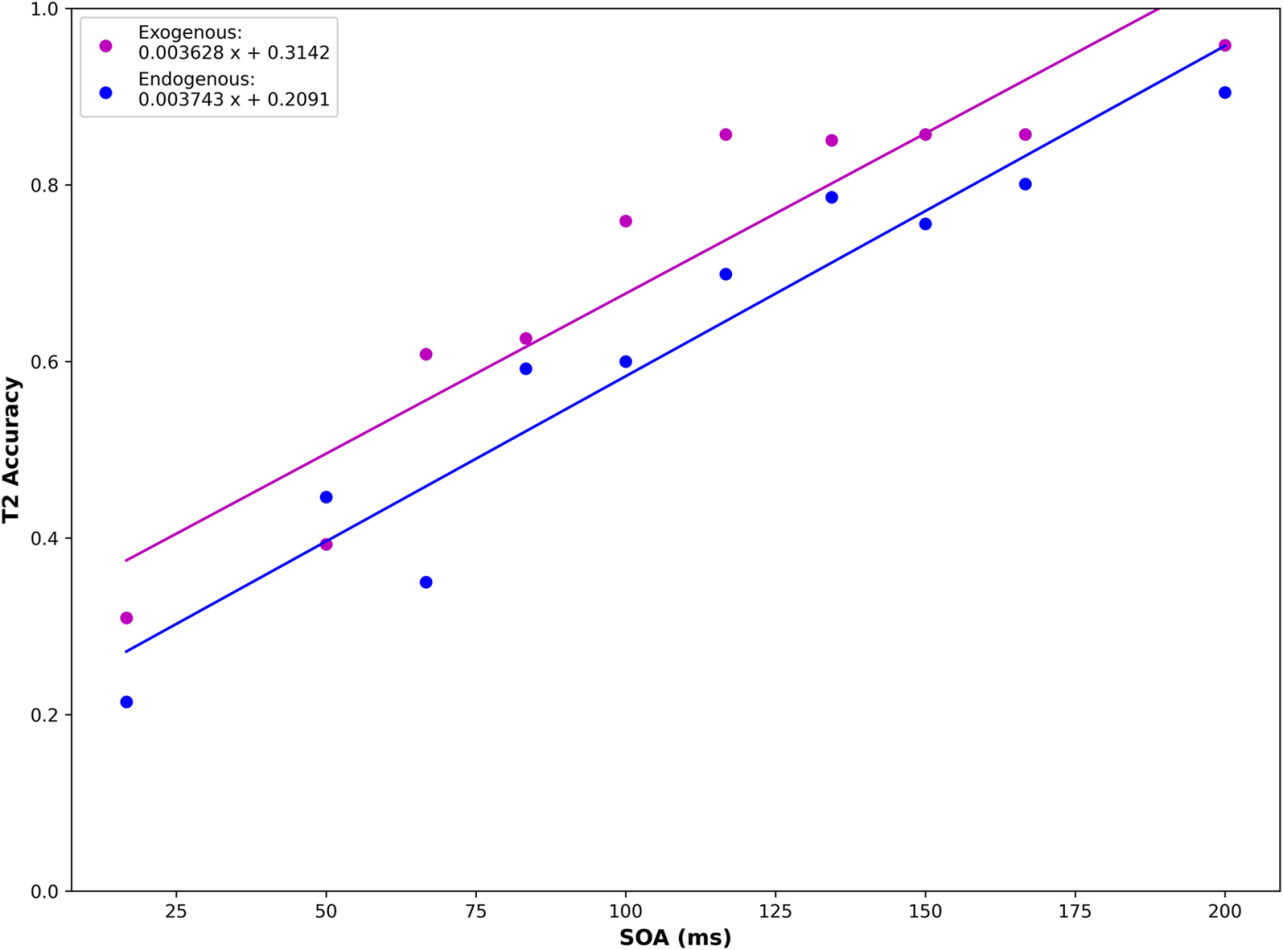
Comparison between exogenous and endogenous lag-1 paradigms reveals a temporal cost for lag-1 sparing in top-down attention. Blue circles (endogenous) and pink circles (exogenous) denote average T2 accuracy (n + 1) across subjects (N = 13) for each SOA. Blue and pink lines represent linear regression.

Prior spatial attention work has suggested that activating top-down attention requires more time than bottom-up attention. To investigate the extent to which this finding holds without spatial shifts of attention, we conducted an experiment comparing T2 accuracy for top-down versus bottom-up cueing of attention. Trials were of identical structure to Experiment 1 in which simply the speed of presentation was varied without fillers (t_image_ = SOA = [16.67–200 ms]; Table 1). However, subjects performed 90 trials of randomly interleaved top-down and bottom-up conditions. The number of trials performed at each SOA was weighted around the middle SOA range [50–150 ms] in order to be more sensitive to potential differences between the top-down versus bottom-up conditions. For conditions testing bottom-up attention, the cue was a large white “X,” a pop-out stimulus that was more likely to trigger an orienting response to engage attention. Results from this experiment revealed higher average T2 accuracy on bottom-up compared to top-down paradigms (Fig. 6). Linear regression demonstrated that top-down and bottom-up processes had similar slopes (Exogenous: 0.0036, Endogenous: 0.0037), but different T2 accuracies for a given SOA (T2 intercept: Exogenous (0.31); Endogenous: 0.21).

## Discussion

The present study sought to understand the temporal dynamics of the top-down engagement of attention in lag-1 sparing. Prior lag-1 sparing work has produced several theories to explain this phenomenon, including capacity-limited models, the attentional gate theory, and the boost and bounce theory^6,13–17^. Other studies have supported the idea that endogenous control of the attentional window may directly impact the ability to accurately identify successive targets^20,21^, and that attentional facilitation from the identification of T1 has a temporal delay^23^. However, the factors that govern these temporal dynamics remained unclear. Here, we implemented an adapted lag-1 sparing RSVP paradigm to minimize the potential confounds of exogenous (“bottom up”) activation. We found that the engagement of attentional facilitation in lag-1 sparing required between 50 and 100 ms. By controlling for stimulus presentation rate versus image duration, we found that capacity-limited perceptual processes were less consequential for accurate T2 identification than simply the time afforded for an attentional boost to engage attention. In other words, the intrinsic dynamics of attentional facilitation appeared to be the primary rate-limiting mechanism for accurate lag-1 performance.

### The lower limit of lag-1 sparing lies between 50 and 100 ms

Experiment 1 demonstrated that the range of latencies for reliable T2 accuracy was between 50 and 100 ms (Fig. 2A). To our knowledge, the present results are the first to identify a minimum time window needed to produce lag-1 sparing of T2 in RSVP. Although the underlying neural mechanisms governing this 50–100 ms range remain unclear, it has been suggested that alpha oscillations may play a critical role in attention^30,31^. Recent AB literature supports the idea that alpha phase entrainment may underlie the AB^32,33^ and that pre-stimulus alpha phase can predict perception in detection-based paradigms or tasks with exogenous cues^30,34,35^. It is therefore possible that an underlying alpha oscillation influences flexible control of endogenously-driven attention in lag-1 sparing. This is roughly consistent with our estimate here (50–100 ms = ~ 10–20 Hz). At SOAs outside of this window, the success of T2 capture may fluctuate based on target presentation aligning with the optimal pre-stimulus alpha phase. Conversely, and in support of prior work^21^, at presentation rates above this temporal threshold, endogenous control process dependent on multiple oscillatory cycles may engage between the presentation of T1 and T2 in the RSVP stream, yielding capture of T2. It is important to note that recent findings from non-RSVP paradigms have related reorientation of voluntary attention to periodic theta oscillations representing frontal-occipital communication and have suggested that 5–7 Hz theta waves correlate with attentional sampling (versus sensory sampling at alpha frequencies)^36–40^. Future research to understand the role of alpha versus theta oscillations in top-down attentional dynamics are necessary. Specifically, it is unclear whether theta, alpha, or oscillations in both frequency bands are involved in aligning the native attentional rhythm to the rate of sequentially presented stimuli, and to what extent these oscillations may coordinate activity across relevant brain regions.

The results from experiment 1 also have implications for models of lag 1 sparing. Several groups have suggested this phenomenon occurs because T1, the initial target stimulus (analogous to the cue in our paradigm), triggers the opening of an attentional gate. The closing of this gate is delayed such that a T2 (analogous to the target image in our paradigm) presented sufficiently close to T1 allows both targets to be processed together^5,6,9,13^. These studies have suggested that temporal succession and shared characteristics are sufficient for lag-1 sparing to occur when the attentional gate is open. However, if this were true, lag-1 sparing should occur even at short SOAs. The present findings demonstrate that T2 accuracy dramatically decreases between SOAs of 100 ms and 50 ms, thus contradicting this idea. Therefore, there must be another factor governing the visual dynamics of lag-1 sparing.

More recently, the boost and bounce theory has suggested that T1 generates an attentional boost that facilitates the processing of T2 and other temporally proximal stimuli^17^. This transient attentional facilitation may also be temporally delayed (approximately 100 ms). Our present findings of high target accuracy at SOA up to 250 ms demonstrate that lag 1 sparing can occur at intervals greater than 100 ms for images (Fig. 2) and alphanumeric stimuli (Supplementary Fig. 3A). Furthermore, the observed low accuracy at short SOAs and the 50–100 ms range for the lower limit of attentional engagement support the boost and bounce theory, suggesting that the recognition of the T1 cue stimulus triggers an attentional boost that allows for capture of T2, and that the temporal delay required to engage boosting processes lies between 50 and 100 ms. Insufficient time between the presentation of T1 and T2 may result in poor T2 identification because these processes have not taken full effect.

### Attentional facilitation in lag-1 sparing is constrained by a temporal delay, not perceptual processes related to stimulus integration or identification

Experiment 2 demonstrated that the rate of stimulus presentation, not stimulus duration, was the limiting factor for accurate T2 identification, with performance accuracy decrements proportional to the change in SOA (Figs. 2D and 3). These results were also replicated in a supplemental set of experiments using alphanumeric characters instead of images in the RSVP stream (Supplemental Fig. 3B,C). This is consistent with the boost and bounce theory and contradicts models of visual processing that focus solely on accumulation of visual information. Based on these results, high T2 performance depends either on having sufficient time to engage the attentional boost or on having enough time to clear “space” in capacity-dependent working memory between presentation of T1 and T2. Results from experiment 4 demonstrated that, at fast SOAs, performance was not limited by capacity-dependent visual processes. Rather, accurate T2 identification was high so long as sufficient time was available to engage attentional resources despite the rapid presentation of additional, intervening images (Fig. 5A,B). This finding supports our hypothesis that the major constraint on lag-1 sparing is the amount of time needed to engage an attentional boost to T2. Lower level perceptual processes—such as accumulation of information about T1 and T2 images in the visual stream (image duration) or the rate of visual input itself (apart from the critical T1 to T2 SOA)—impact accurate T2 identification less than the intrinsic dynamics of lag-1 attention engagement. Taken together, these results provide strong support for the boost and bounce model as an explanation for lag-1 sparing, rather than capacity-limited visual processing or attentional gating theories.

Interestingly, when comparing the time necessary to trigger lag-1 sparing by bottom-up verses top-down processes, we found higher average T2 accuracy on bottom-up compared to top-down paradigms for a given SOA, which was supported by linear regression analysis. These data suggest that lag 1 sparing in bottom-up versus bottom-up paradigms may invoke similar visual processing mechanisms but has a distinct time course. This finding is in line with prior research showing that reflexive orienting to peripheral cues leads to faster responses than the voluntary orienting of attention^13,41,42^. This difference may simply reflect the extra time needed to attend to T1 (not an exogenous, pop-out stimulus) in the top-down condition, compared to the bottom-up trials where the pop-out T1 processing is automatic. Exogenous (“pop-out”) stimuli are also typically simpler and may be sufficiently processed earlier in the ventral visual stream, whereas more subtle images that do not produce a “pop-out” effect likely need to be processed through further stages in the visual stream.

Although we found that slower stimulus presentation rate was associated with accurate T2 identification (Fig. 3), there is likely a minimum level of exposure to a stimulus that is necessary to generate a suitable representation for that object to be compared to the contents of working memory. The present study tested image durations only as short as 16.67 ms. A systematic investigation varying image duration at smaller t_image_ values will be necessary to estimate a threshold for stimulus duration.

Interestingly, the average T2 performance rate for the t_image_ = 50 ms condition in Experiment 2A (20.53%; Supplementary Fig. 1A) was significantly lower than for the identical condition in Experiment 1 (*p* = 0.0004, 43.33%; Fig. 2B). The presence of interleaved filler and non-filler trials in Experiment 2A may explain this effect. Specifically, if a sufficient proportion of trials within an experiment block contained fillers, subjects might have been able to adapt visual processes or attentional dynamics to accommodate for the interleaved distractor (“filler”). This adaptation may have led to performance on trials where no filler was present to be lower than prior performance on identical trials in blocks without fillers due to an expectancy effect^21^. Therefore, the proportion of trials with vs. without fillers should influence the use or success of this altered attentional strategy. This possibility will be tested in future studies.

## Conclusion

The present experiments provide convergent evidence that the intrinsic temporal dynamics of attention constrain lag-1 sparing, consistent with the “boost and bounce” hypothesis, with a lower limit for the boost latency between 50 and 100 ms. Lag 1 sparing limit was not as dependent upon capacity-limited visual processing or a requirement for sufficient accumulation of visual information, but rather was a function of the amount of time available between T1 and T2 for the attentional engagement to occur. Additionally, top-down versus bottom-up mechanisms for engaging attention in lag 1 sparing may be similar, but operate on different time courses. These results provide new insight into the fundamental constraints of the human visual system when selecting desired information from an inundation of visual inputs from our environment.

## Methods

### Task

The RSVP paradigm challenges visual processing by presenting multiple images rapidly in the same visual field location^1^. Most simply, our task required subjects to identify a pre-specified cue image in the RSVP stream and report the image immediately following. More specifically, we required subjects to identify, within a 10-image RSVP stream, a trial-unique cue image (T1), and then to encode the image immediately following that cue (T2) for later selection during a 6-alternative forced-choice phase at the end of each trial (Fig. 1). The 5 distractors in the choice phase were the remaining non-T1, non-T2 images used in that trial, and excluded the first, second and last images of the RSVP sequence (those were not eligible to be targets, see below, to reduce primacy and recency biases). Subjects indicated their choices by moving a joystick-controlled cursor to one of the six image options during the choice phase. Chance performance was 16.7%. In addition to appearing within the RSVP sequence, the T1 cue image was presented at the beginning of each trial, separately, to inform subjects of its identity prior to the RSVP sequence. Importantly, the cue image was itself the T1 image in the RSVP stream. Presentation rate was termed Stimulus Onset Asynchrony (SOA)—the duration of time between the onset of one RSVP image and the onset of the next image in the stream, notwithstanding the presence of any “filler” (see below).

Figure 1 illustrates the structure of our RSVP task (SOA = t_image_ = 250 ms). Subjects were first presented with the T1 cue centered against a black background for 3000 ms followed by a 1000 ms delay during which just the fixation spot was present. After this delay, a series of 10 images were displayed in rapid succession at the same location in visual space. The SOA and the duration of each image (t_image_) in the RSVP stream varied by condition in each block but remained consistent within a particular trial. Conditions were randomly interleaved within blocks. T1 position was randomly assigned to appear between the second and eighth image, inclusive, and so T2 could appear at positions 3–9. During trials with “fillers,” each RSVP image was followed by a 6.54° (300 × 300 pixel) square in the same position with the same average luminance as the preceding image. Filler color was assigned as the average RGB value of the image directly preceding it in the RSVP stream. Importantly, fillers are not acting as visual masks. Rather, they simply serve as a control for low level visual stimulation. Filler duration (ISI) was defined as the remaining time when the image duration was subtracted from the SOA (ISI = SOA − t_image_). The use of fillers allowed for independent control of image duration and presentation rate. For supplemental Block A, the T1 cue image for conditions testing bottom-up attention was a large, white “X” (equivalent aspect ratio to the 6.54° pictures) against the black background, and the T1 cue for conditions testing top-down attention was a randomly selected image (see “Experimental setup” section above). T2 for both the bottom-up and top-down conditions was the image immediately following the T1 stimulus in the RSVP stream. Supplemental Experiments 1, 2A, and 2B used alphanumeric characters for the cue and RSVP stream instead of image stimuli. Each alphanumeric stimulus consisted of a white letter or number against a gray square background (6.54°, equivalent aspect ratio to images).

Importantly, during the choice phase, T1 (cue in position “n”) was never an available option, and T2 (lag 1 image) was always an option. The locations of the six image options were randomly shuffled such that the image appearing at any particular index during the RSVP stream (i.e. n − 1, n + 1, n + 2, etc.) was not consistently presented at the same choice location across trials. A circular arrangement of choices ensured that each image option had the same eccentricity and thus comparable visibility. Upon selection, a colored box indicating a correct (green) or incorrect (red) response was displayed around the chosen image. Subjects were given a maximum of 30,000 ms to choose the target; the vast majority of response times were far below this limit. During the block comparing trials with exogenous vs. endogenous cues, the maximum time for target choice was decreased to 10,000 ms. When a response was recorded or the time limit was reached, whichever came first, a 2000 ms intertrial interval (ITI) ensued.

### Participants

A total of 42 participants (21 male, 19 female, 2 other, mean age = 20.29 years, range 18–32 years) were recruited from the Brown University and broader Rhode Island communities. At each visit, subjects were given 10 trials to acclimate them to the RSVP task. Acclimation trials had SOA = 250 ms and followed the same format as trials in each experiment block (Fig. 1). Following the acclimation trials, subjects performed at least one of 5 experiments. Experiment 1 consisted of 60 trials, both Experiment blocks 2A and 2B involved 50 trials, and Experiment Blocks 3A, 3B, and 4 were each comprised of 30 trials. Experiment 5, which compared top-down and bottom-up attention, consisted of 90 trials (Table 1). A maximum of 290 trials were performed per session. If multiple experiment blocks (e.g., 2A and 2B) were completed within a testing session, participants were given breaks between sets. Each subject provided written informed consent prior to beginning the study and had normal or corrected-to-normal vision. The study was approved by the Institutional Review Board at Rhode Island Hospital, and all experiments were performed in accordance with the guidelines and regulations set by this institution.

### Experimental setup

Stimuli were presented on a 27-inch LED monitor (resolution 1920 × 1080 pixels, refresh rate 60 Hz) using the NIMH MonkeyLogic software for MATLAB^43,44^. Subjects were seated approximately 57 cm from the monitor. Pictures were obtained from the Common Objects in Context (COCO) database, which includes photographs of naturalistic scenes, people, animals, and objects^45^. Each image subtended 6.54° (300 × 300 pixels). Filler squares of the same size were generated for each picture using a custom python script that assigned the color based on the average RGB value of the corresponding image. All experiments were performed in a well-lit room.

### Statistical analysis

Summary statistics on the study population were obtained. Subjects were required to choose the image in the correct position within the sequence (i.e., the target presented at position n + 1, where n is the cue). T2 performance accuracy was calculated as the number of trials where subjects chose the correct image divided by the total trials where they made an answer selection. To analyze incorrect choices, we counted the number of times subjects selected each different image option (e.g. n + 2) and normalized this by the number of trials in which the RSVP stream image at that position was available in the 6-alternative forced choice answer phase. Comparison of accuracy between two groups was performed using one-sample independent t-test for equal variances and Welch’s t-test for unequal variances. One-way ANOVA was used to investigate differences in performance between 3 or more sets of conditions. Linear regression was used to fit the n + 1 accuracy for all SOAs. Analyses was first performed by subject, then as a group. The threshold range for attentional engagement was determined by identifying the SOA values where T2 accuracy switched from above chance to not significantly different than chance. All analyses were performed in Matlab Version R2020b (MathWorks, Natick, MA) and Python Version 3.8.3 (Python Software Foundation, Beaverton, OR).

## Supplementary Information

Supplementary Figures.

## Data Availability

De-identified datasets for the current study are available from the corresponding author on reasonable request.
