# Supplementary material for: The temporal cost of deploying attention limits accurate target identification in rapid serial visual presentation
Source: Sci Rep. 2023 Mar 3;13:3590. doi: 10.1038/s41598-023-30748-z (PMC9984373; doi:10.1038/s41598-023-30748-z)

Supplementary Figure 1: Average hit rate (y-axis) by distance from the target in the RSVP stream (x-axis) for each SOA. Number of trials represented by the data in each subplot is shown in parenthesis. **(A)** Presentation rate (SOA) varied [50-250ms], but image duration (t_image_) was held constant at 50ms. **(B)** SOA was held constant at 250ms, but t_image_ varied [50-250ms]


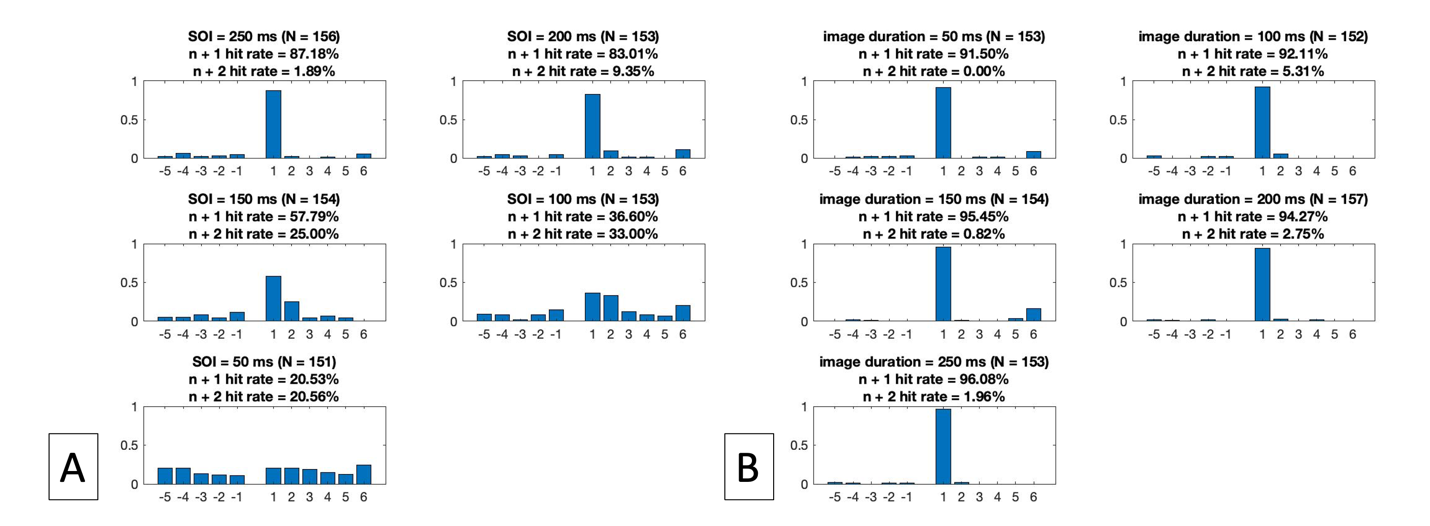


Supplementary Figure 2: Average hit rate (y-axis) by distance from the target in the RSVP stream (x-axis) for each image duration (t_image_). SOA = 50ms for all trials, image duration (t_image_) varied between [16.67-50ms]. Number of trials represented by the data in each subplot is shown in parenthesis.


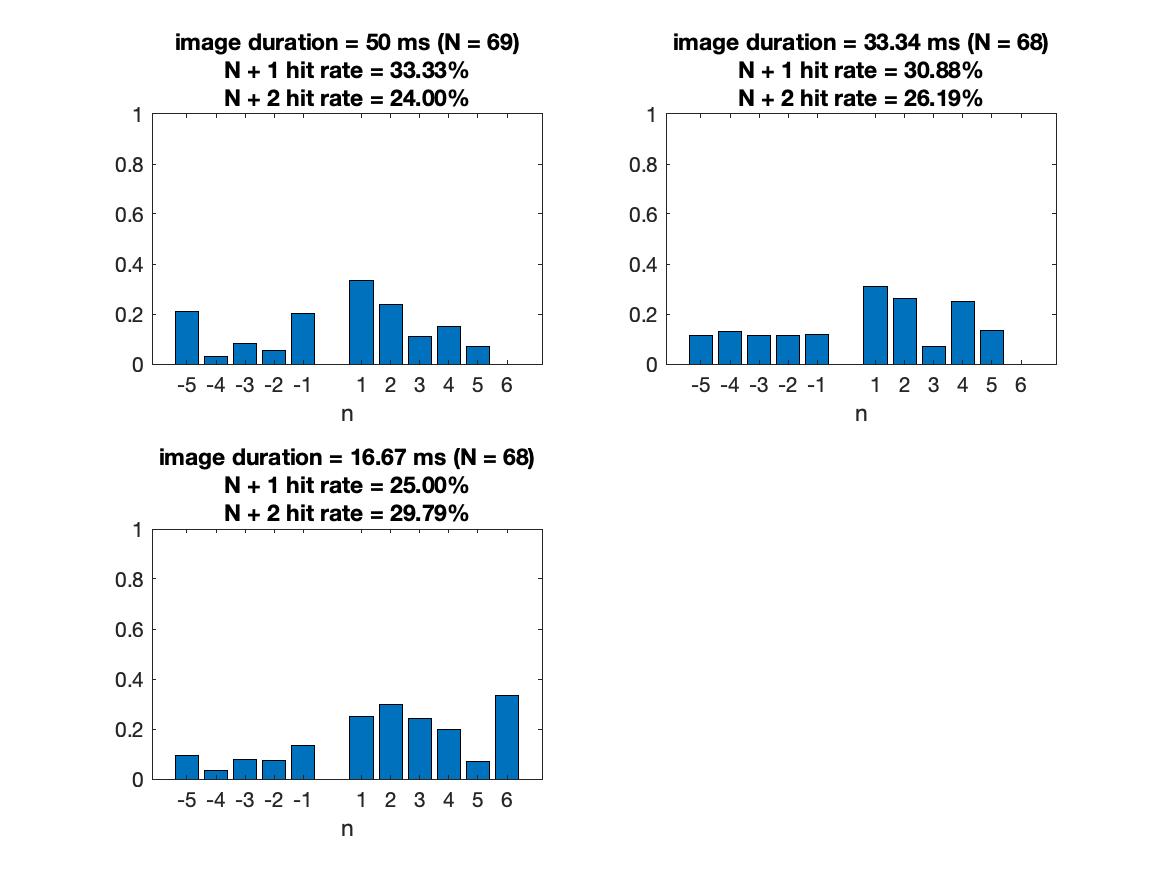


Supplementary Figure 3**:** Average target (n+1) hit rate for each SOA [16.67-250ms] shows increasing accuracy as the SOA increases for alphanumeric characters. Linear regression demonstrated a linear relationship where T2 accuracy = 0.0031*SOA + 0.26.


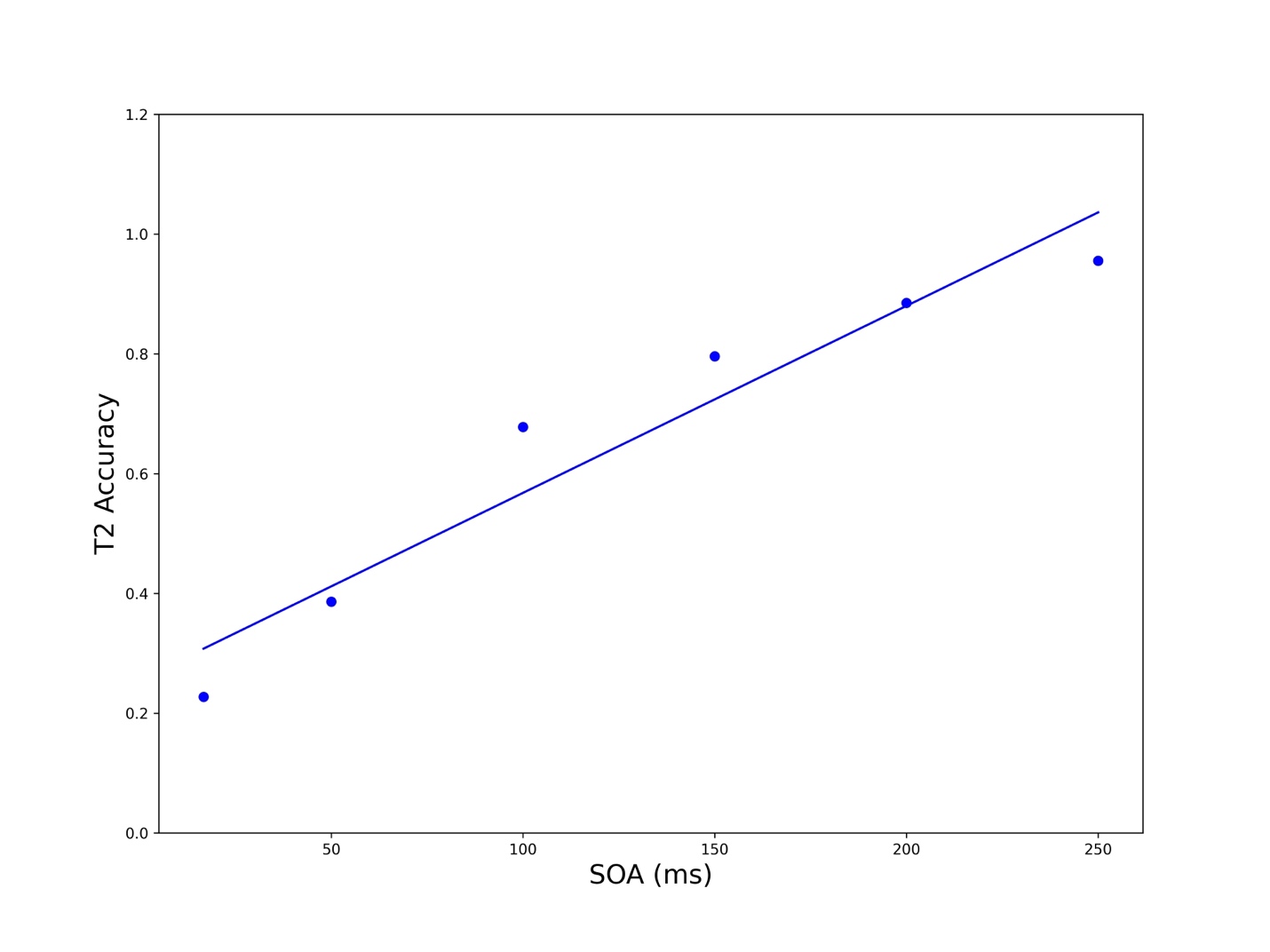


Supplementary Figure 4: Average hit rate (y-axis) by distance from the target in the RSVP stream (x-axis) for each SOA using alphanumeric characters. N = number of trials represented by the data in each subplot. **(A)** Presentation rate (SOA) varied [50-250ms], but image duration (t_image_) was held constant at 50ms. **(B)** SOA was held constant at 250ms, but t_image_ varied [50-250ms]


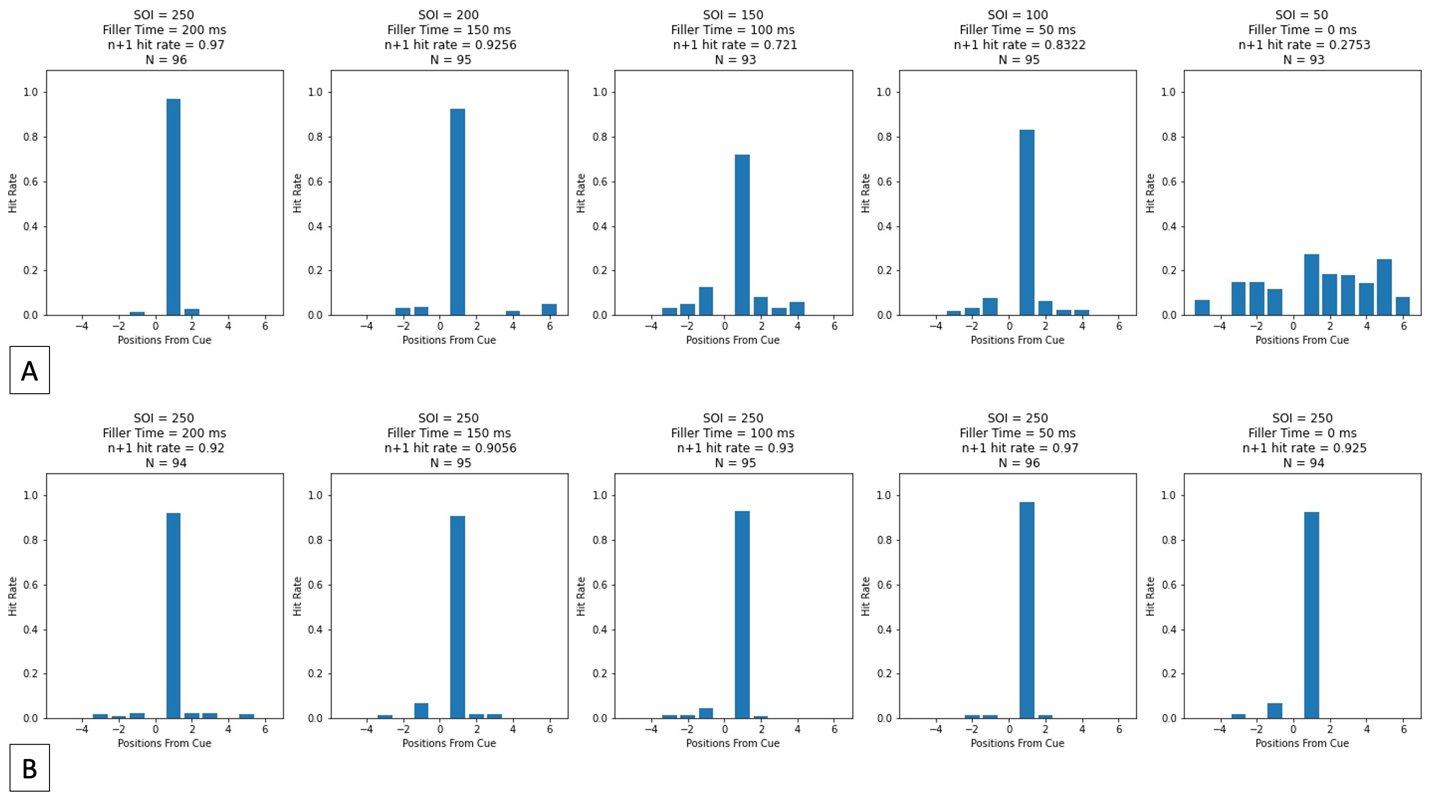

Supplement: Supplementary file 1 — Supplementary Figures. [file 41598_2023_30748_MOESM1_ESM.docx]
